# Sharing Medicine: The Candidacy of Medicines and Other Household Items for Sharing, Dominican Republic

**DOI:** 10.1371/journal.pone.0101007

**Published:** 2014-06-27

**Authors:** Michael N. Dohn, Hugo Pilkington

**Affiliations:** 1 Community Health, Clínica Episcopal Esperanza y Caridad, San Pedro de Macorís, Dominican Republic; 2 Society of Anglican Missionaries and Senders, Ambridge, Pennsylvania, United States of America; 3 Geography Department, Université Paris 8 Vincennes-Saint-Denis, Saint-Denis, France; 4 London School of Hygiene and Tropical Medicine, London, United Kingdom; Indiana University and Moi University, United States of America

## Abstract

**Background:**

People share medicines and problems can result from this behavior. Successful interventions to change sharing behavior will require understanding people’s motives and purposes for sharing medicines. Better information about how medicines fit into the gifting and reciprocity system could be useful in designing interventions to modify medicine sharing behavior. However, it is uncertain how people situate medicines among other items that might be shared. This investigation is a descriptive study of how people sort medicines and other shareable items.

**Methods and Findings:**

This study in the Dominican Republic examined how a convenience sample (31 people) sorted medicines and rated their shareability in relation to other common household items. We used non-metric multidimensional scaling to produce association maps in which the distances between items offer a visual representation of the collective opinion of the participants regarding the relationships among the items. In addition, from a pile sort constrained by four categories of whether sharing or loaning the item was acceptable (on a scale from not shareable to very shareable), we assessed the degree to which the participants rated the medicines as shareable compared to other items. Participants consistently grouped medicines together in all pile sort activities; yet, medicines were mixed with other items when rated by their candidacy to be shared. Compared to the other items, participants had more variability of opinion as to whether medicines should be shared.

**Conclusions:**

People think of medicines as a distinct group, suggesting that interventions might be designed to apply to medicines as a group. People’s differing opinions as to whether it was appropriate to share medicines imply a degree of uncertainty or ambiguity that health promotion interventions might exploit to alter attitudes and behaviors. These findings have implications for the design of health promotion interventions to impact medicine sharing behavior.

## Introduction

Medicines are “universally popular” [1] and people share them [2]. A third of adults have shared medicines [3]–[7] and sharing is seen across age groups and cultures [3]–[12].

The worth and power of medicines relates to their materiality – their “thinginess” [2] – which allows medicines to circulate and to acquire symbolic, metaphoric, and metonymic associations [13]. People share material things, including medicines [2], [14]. Medicines, along with other things, enter into a social gifting and reciprocity system [15]–[17] in which people exchange things to reinforce and maintain social relationships [18].

Medicines escape from their original biomedical context through a variety of sanctioned activities (such as distribution to users with medically-defined problems) and unsanctioned routes (such as drug diversion) [19]. Once medicines are removed from their original context, altered meanings may be imputed to them and the medicines may be utilized for different purposes than the original intent [2], [13], [20], [21].

Medicine sharing can result in multiple problems, ranging from mild gastrointestinal upset, to unintentional fetal exposure to drugs, to problems of epidemiological concern such as antimicrobial resistance [4], [8], [22]. Other concerns include the possibility of incorrect use after medicines become separated from their instructions and warnings; incomplete treatment; drug interactions; delays in care; increased adverse effects or poisoning; addiction with associated personal and social costs; impacts on research; and effects on post-approval drug adverse event surveillance [6], [23].

People may share medicines to avoid costs, for convenience, when lacking access to care, and when not feeling sick enough to seek a consultation [3], [5], [22]. However, medicines may also assume various associations and meanings which may provide people’s motivations to share [1], [13], [24]. Sharing medicine can be a sign of relationship or relatedness. Administering a medicine can be a sign of caring or of the fulfillment of a parental obligation, publicly demonstrating that the parent is loving and responsible [1], [24]. Members of medical short-term mission teams visiting the Dominican Republic have represented the medicines they distribute as symbols of their caring, largesse, liberality, or faith. In contrast, Dominicans have sometimes understood these medicines as symbols of the visitors’ wealth, their naivety when the medicines are inappropriate for the prevalent illnesses, or their disrespect when the medicines are obviously cast-offs (such as expired pharmaceuticals or excess physician samples). The possibilities for imputed meanings are diverse and broad.

Understanding the reasons underlying people’s decisions is essential to designing effective health promotion interventions [1], [25]. Understanding popular beliefs regarding self-medication and the use of medicines becomes particularly important when promoting the rational use of medicines [26]. Additional insight into medicine sharing behavior – the how, why and when of medicine sharing – could inform efforts to develop effective interventions to avoid the problems encountered when medicines are shared.

In this study, medicines and commercial medicines refer to pharmaceuticals based on biomedical knowledge that are produced as a commercial product and packaged for retail sale by a recognized company or corporation [1]. Commercial medicines may include both prescription and over-the-counter preparations. Home remedies, herbal remedies, and nutritional remedies are considered part of the *materia medica* of folk medicine.

Interventions to increase the rational use of medicines could be improved by understanding the viewpoint of patients [20], and in this case those who share medicines. However, it is uncertain how medicines relate to other items that may enter the gifting and reciprocity system. In this study we assessed the shareability of some commercially produced medicines (both prescription and over-the-counter medicines) compared to other objects that could be shared between families, friends, and neighbors.

## Methods

### Ethics Statement

All participants signed written informed consent forms prior to participation. All aspects of this study, including the written informed consent and documentation process, were approved by the ethical review committee of Clínica Episcopal Esperanza y Caridad and by the Combined Risk and Ethics committee of the London School of Hygiene and Tropical Medicine.

### Participants

A convenience sample of at least 30 subjects was planned (30 subjects were required to generate reliable results from semantic differential scales that were in the original thesis plan). Inclusion criteria included: age 18 years or older; Dominican national; and able to recognize items from pictures and written descriptions on the cards to be used for the sorting and ranking exercises. Exclusion criteria included: unable or unwilling to provide informed consent; inability to understand or complete the exercises; medical professional or health care worker; and first or second generation immigrant. Immigrants were excluded as they might not reflect Dominican cultural understandings. Furthermore, family and household members of people who had completed the activities were subsequently excluded. Initial participants were identified in three separate geographical areas. One area was within the city limits, one area was a small adjoining municipality, and the other area was in a mixed urban and rural area. Initial identification of potential participants occurred with the assistance of a person in each of those communities who had been involved with past research activities [27], [28]. These people (two school administrators and a patient peer counselor) understood the basics of research and informed consent, and were asked to identify possible participants who could complete a pile sort exercise. Initial participants were suggested by these intermediaries. Subsequent potential participants were referred by these intermediaries and by people who had completed the study, causing some snowball sampling characteristics.

### Data Collection

Data collection was through individual encounters (about 30 minutes in length). After completing the informed consent process, participants’ provided demographic information (sex, age, marital status), self-reported health conditions and diagnoses, socio-economic status (educational level and housing information), and whether the participant had ever shared medicines with anyone.

Participants completed three pile sort exercises. Afterwards, brief conversations occurred with participants (for instance, concerning the rationale behind their pile sort groupings).

In the first free pile sort (sort 1), participants were given a set of 44 randomized cards with names and photos of medicines and other household items that could be shared. The 34 non-medicine items were chosen from among items identified during a previous free listing exercise related to things that people could share. Participants were asked to group the cards into piles. For this and all pile sorts, the number of piles must have been at least two (all cards could not be grouped together) and less than the total number of cards (at least one association had to emerge). The piles of cards were collected and an association matrix (44 by 44 in this case) was generated for each participant. In each participant’s association matrix, we recorded every instance in which an item was grouped with some other item in a specific pile. For example, in a pile of three items (n = 3), there would be six associations (associations = n^2^ −n) recorded in the matrix for that one specific pile (a with b and c; b with a and c; and c with a and b). The individual participants’ matrices were combined in a summary matrix that gave the percentage of all pile sorts in which any two items were placed in the same pile. Non-metric multidimensional scaling analysis uses the summary matrix to construct an association map in which the distances between items offer a visual representation of the collective opinion of the participants regarding the relationships among the items [29].

In the constrained pile sort (sort 2), participants were asked to sort the same 44 items on a four-category, horizontal, analogue scale based on the item’s candidacy for sharing (Figure S1 in File S1). The scale was sufficiently large that participants could place the pile sort cards directly on the categories on the scale. The four categories were assigned numerical values for evaluation (from 0 for “inappropriate to share” to 3 for “very appropriate to share”). The values for each category on the scale were used to calculate the mean shareability score and its standard deviation (SD) for each item. The scores were used in the non-parametric analyses of ordinal data to compare the group of medicines to the other items. This activity also generated groups for non-metric multidimensional scaling analysis as in the other pile sorts.

For the free pile sort of 33 items from the local *materia medica* (sort 3), participants were given a set of 33 randomized cards with names and photos of pharmaceuticals, over-the-counter medicines, and home remedies related to five health conditions (headache, hypertension, intestinal parasites, anemia, and a chest cold or “tight breathing”) as well as several “unclassified” items (Table S1 in File S1). The home remedies were chosen from among those listed during a past free listing exercise. Participants sorted the cards into piles as previously described, and the results were recorded using 33 by 33 matrices.

### Statistics

Statistical analysis of the data was conducted using both descriptive and analytical methods [30], [31]. Analysis of continuous data was by t-test for two groups and one-way analysis of variance for multiple groups. Ordinal data was compared for two groups by Wilcoxon rank sum test and for multiple groups by the Kruskal-Wallis test. Categorical data was analyzed using contingency table methods.

For pile sort 1, the individuals’ pile sort data were combined as described by Bernard [32] using a spread sheet program (Excel 2010, Microsoft, Redmond, Washington, USA) to generate a summary matrix showing the percentage of all sorts in which any two items were in the same pile. Summary matrices were also generated for sort 2 and sort 3. Non-metric multidimensional scaling analysis was applied to the three summary matrices using a public domain anthropology analytical program (Anthropac 4.983, Analytic Technologies, Natick, Massachusetts, USA). Non-metric multidimensional scaling analysis uses the summary matrix to construct an association map in which the distances between items offer a visual representation of the collective opinion of the participants regarding the relationships among the items.

The numerical values associated with the four categories of shareability on the analogue scale in the constrained pile sort (sort 2) were used to generate a mean shareability score and its SD for each of the 44 items.

A commercial statistics program (Stata/IC 10, StataCorp, College Station, Texas, USA) was used for the standard statistical analyses. Statistical results with probability (P) values less than 0.05 were considered significant. Overall Type I error was controlled to a level of 0.05 for multiple pair wise comparisons.

## Results

During the two weeks from 25 April through 8 May, 2012, 33 people were invited to participate in this study; 31 people accepted and gave consent. The convenience sample included a mixed population (Table 1). Ages ranged from 19 to 56 years (mean 37.6, median 38, SD 8.7) and were not different by sex (P = 0.58).

**Table 1:** Characteristics of study participants (n = 31).

Characteristic	Categories	Number of participants
Sex	Female	26
	Male	5
Community	Urban neighborhood	19
	Marginal urban community	12
Marital status	Single	18
	Married	9
	Common law marriage	4
Educational level	Less than primary	1
	Completed primary	6
	Completed secondary	12
	At least some university study	12
Taking medicines	Yes	13
	No	18

Thirteen people had self-reported chronic health conditions and were taking medications, including three participants with human immunodeficiency virus (HIV) infection who had all received treatment literacy training. These three people spontaneously expressed opposition to medicine sharing. For example, after completing the activities, one of them delivered an extemporaneous, emotional admonishment against sharing medicines in a lecturing tone, including finger pointing.

The results of two pile sort exercises from the first participant were lost prior to analysis (thus the results from sort 1 and sort 3 include only 30 participants).

**Figure 1:**
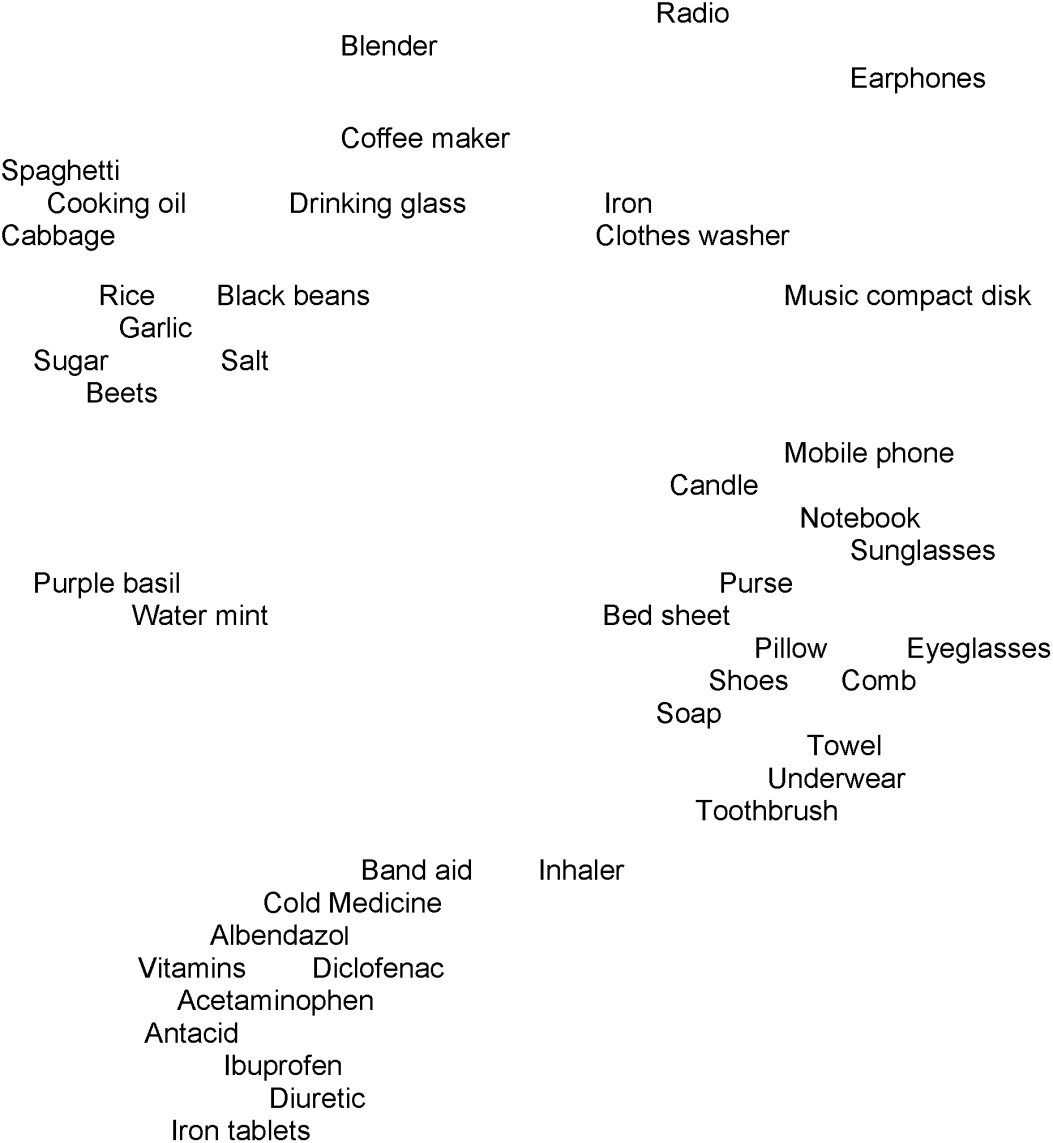
Pile sort association map of 44 items that could be shared (sort 1). This association map shows the groupings of 44 items that could be shared that emerged from the free pile sort (n = 30 sorts). Some items have been shifted to avoid overlapping text.

Non-metric multidimensional scaling analysis produces an association map in which the distances between items offer a visual representation of the collective opinion of the participants regarding the relationships among the items. The association map of the free pile sort activity of 44 items that could be shared suggested a tight grouping of “medicines” (near the bottom of Figure 1) with looser groups of what might be called “foods” and “personal items” (often described by participants as “my things” or “things I keep in the bathroom”), as well as other scattered items (Tables S2, S3 and S4 in File S1). The group of “medicines” had the closest spatial relationship to the group of “personal items”.

**Figure 2:**
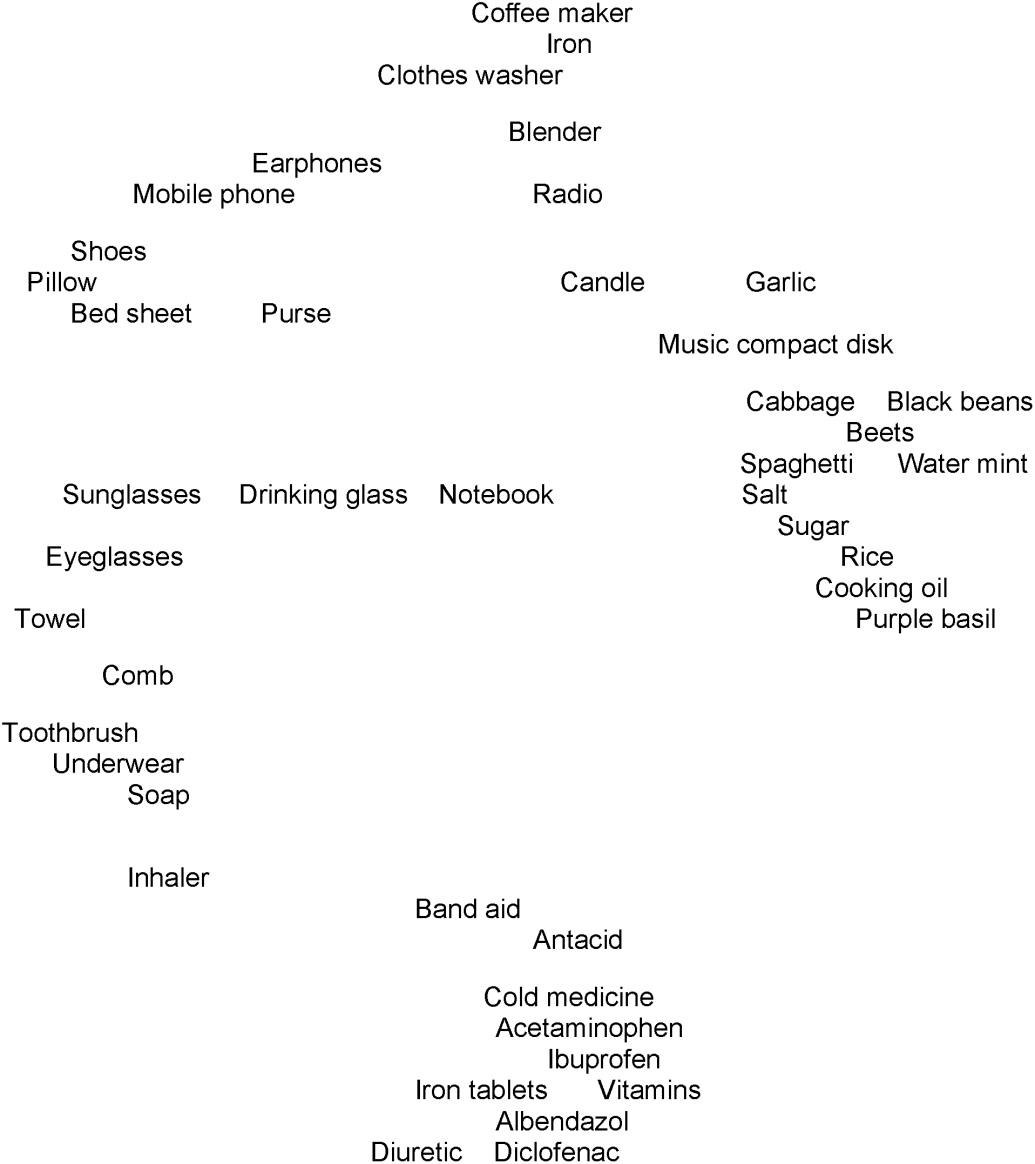
Pile sort association map of 44 items when constrained by shareability (sort 2). This association map shows the groupings of 44 items that could be shared when the pile sort was constrained to four categories of shareability (n = 31 sorts). Some items have been shifted to avoid overlapping text.

Groupings from the constrained pile sort activity suggested that people maintained the “medicine” and “food” groups when sharing (Figure 2). Items from the original “personal items” group in the first pile sort were more dispersed, though generally continuing to be more closely associated with “medicines” than were the other items.

The items’ mean shareability scores ranged from 0 to 2.64. Values could range from 0 to 3 (from “not appropriate to share” to “very appropriate to share”, respectively). Higher mean shareability scores indicate greater candidacy for sharing activity. There was no difference between the shareability scores of the ten commercial medicines and the other 34 items (P = 0.54) (Table 2).

**Table 2:** Individual mean “shareability scores” of 44 items in the constrained pile sort.^a^

Black beans	2.64	Salt	2.19	Band aid	1.68	Bed sheet	0.90
Water mint	2.55	Diclofenac^b^	1.90	Iron tablets^b^	1.68	Mobile phone	0.84
Rice	2.55	Cold medicine^b^	1.90	Iron	1.61	Sun glasses	0.81
Spaghetti	2.52	Ibuprofen^b^	1.87	Notebook	1.61	Pillow	0.74
Beets	2.48	Albendazole^b^	1.84	Blender	1.61	Inhaler^b^	0.55
Purple basil	2.45	Antacid^b^	1.81	Clothes washer	1.58	Eye glasses	0.52
Sugar	2.45	Acetaminophen^b^	1.81	Diuretic^b^	1.52	Hair comb	0.48
Cabbage	2.45	Candle	1.77	Ear phones	1.29	Soap	0.42
Garlic	2.39	Vitamins^b^	1.77	Drinking glass	1.13	Towel	0.16
Cooking oil	2.26	Coffee maker	1.71	Shoes	0.97	Underwear	0.06
Music CD	2.19	Radio	1.71	Purse	0.97	Tooth brush	0.00

a Items are arranged by their mean shareability scores from the most shareable (highest values) to the least (lowest values), top to bottom in sequential columns.

b Indicates a commercial pharmaceutical.

The SD’s of the mean shareability scores of the ten commercial pharmaceuticals were generally larger than the SD’s of the other items (P = 0.0007) (Table 3). The SD is a measure of variance; the larger the SD, the less agreement among the study participants on the candidacy for sharing of a particular item.

**Table 3:** Items arranged by the SD of their shareability scores.^a^

Tooth brush	0.00	Garlic	0.80	Shoes	1.02	Notebook	1.15
Underwear	0.25	Spaghetti	0.81	Ear phones	1.04	Vitamins^b^	1.15
Towel	0.37	Hair comb	0.81	Iron	1.05	Albendazole^b^	1.16
Black beans	0.66	Cooking oil	0.82	Salt	1.08	Band aid	1.17
Water mint	0.68	Music CD	0.83	Clothes washer	1.09	Iron tablets^b^	1.17
Purple basil	0.72	Soap	0.88	Purse	1.11	Acetaminophen^b^	1.17
Rice	0.72	Inhaler^b^	0.90	Candle	1.12	Blender	1.17
Eye glasses	0.72	Sun glasses	0.91	Drinking glass	1.12	Ibuprofen^b^	1.18
Sugar	0.77	Pillow	0.93	Diuretic^b^	1.12	Coffee maker	1.19
Cabbage	0.77	Mobile phone	0.93	Diclofenac^b^	1.14	Radio	1.19
Beets	0.77	Bed sheet	0.98	Antacid^b^	1.14	Cold medicine^b^	1.25

a Items are arranged by the SD’s of their mean shareability scores from top to bottom in sequential columns, from those with the most agreement of opinion on their shareability (lowest SD’s) to those with more variability of opinion (higher SD’s).

b Indicates a commercial medicine.

**Figure 3:**
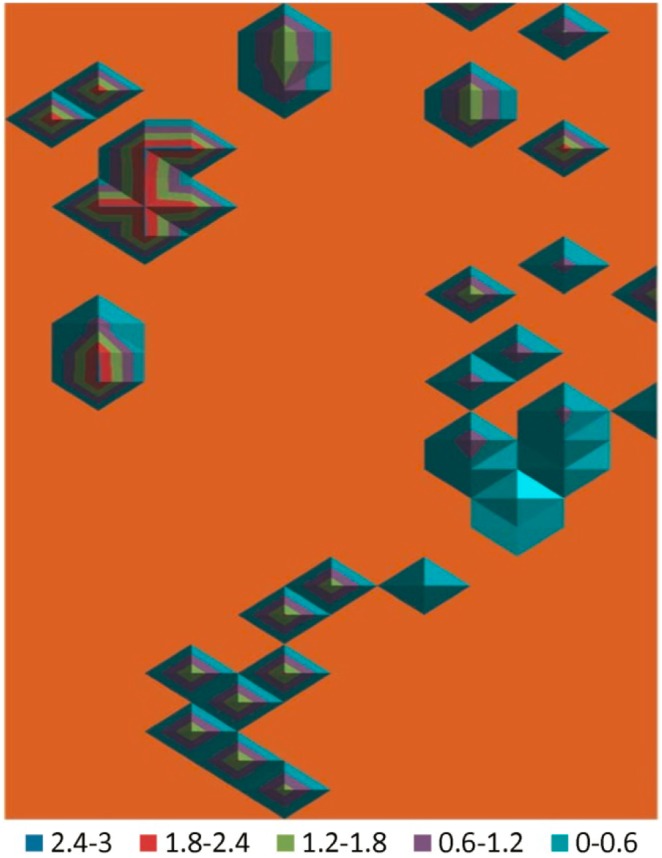
Contour map of shareability scores. Items’ shareability scores are oriented as the items appear in Figure 1. The contour map suggests that items that were grouped together in the free pile sort have similar shareability scores.

Substituting the mean shareability scores for the names of the items in the association map for the free pile sort (sort 1 shown in Figure 1) allowed the construction of a contour graph of the items’ shareability scores (Figure 3). The contour map uses a three-dimensional color “pyramid” over each item, the height and color of which shows the value of that item’s shareability score as shown in the key at the bottom of the figure. The contour map suggested that the shareability scores were similar for items in the same groupings. For example, the “medicines” clustered at the bottom of the graph show colors extending from a blue base through a purple layer and terminating with a green peak. In contrast, the “personal items” along the central right edge are mostly all blue or have a small purple peak, indicating lower candidacy for sharing. The values of the shareability scores for the medicines (grouped toward the bottom in the figure) were generally intermediate between the more shareable food items (clustered toward the upper left corner) and the less shareable personal items (grouped along the middle of the right side).

Results of the free pile sort of 33 items from the local *materia medica* suggested that people group commercial medicines separately from the various home remedies (Figure S2 in File S1). Overall people did not sort these things according to their associated health uses (Figure S3 in File S1).

When asked about the rationale behind the sets of things they had grouped together, most participants did not articulate a rationale for their groupings. Some participants did offer labels for their groups, such as: herbs, medicines, kitchen things, bathroom things, etc. Labels could not be easily collated as different participants put different things into different numbers of groups.

Responses to whether or not participants had previously shared medicines were not informative. All participants had previously shared medicines given the very broad understanding of “medicine” in this study.

## Discussion

This study collected information that could provide insight into how people view medicines and relate them to a selection of other items that could be shared with family, friends, or neighbors. The focus in this study was the metaphoric associations among the medicines and other common household items. This study did not attempt to define metaphoric relationships between medicines and the perceptions or characteristics of diseases – the more commonly recognized realm of metaphoric associations [13].

People grouped medicines together in sort 1 and sort 2, without much evidence that they identified any similarities (metaphoric associations) with the other items. During the sorting activity constrained by sharing categories, participants continued to group medicines as a distinct group of things. These results imply that people may share to some extent the biomedical perspective of the professional sector of the medical system that medicines are “different” or “special” [33]. In the pile sort constrained by shareability, groupings of items tended to be the same as the groups observed in the free pile sort. These results suggest that the apparent categories from the first pile sort (“medicines”, “food”, and “personal items”) may correlate with a sense of ownership or privacy or some other quality related to sharing.

Amongst the medicines, only the respiratory inhaler was positioned apart from the group of “medicines” and toward the “personal items” (Figures 1 and 2). The inhaler had the lowest candidacy for sharing of all the medicines, ranking between “eyeglasses” and “pillow” for shareability. It also had the lowest variance of opinion among the medicines concerning its shareability. “Band aid” appears in proximity to the medicine group. While band aids are not medicines, they are a commercial health product. Similar to “inhaler”, “band aid” occupied an intermediate position between the “medicines” and “personal items” groups. People may see characteristics of inhalers and band aids that are more like the “personal items”. Possibly the intimacy of use (placing an inhaler in your mouth to deliver a mist to the lungs or applying a band aid to one’s skin where it becomes part of your covering for a time) or perhaps the physical manipulation and mechanical properties of the items resemble characteristics of the “personal items”.

The categorization of commercial medicines as a distinct group is not a universal viewpoint. For example, the East African Kiswahili term *dawa* encompasses a complex social and cultural grouping of things that can produce changes with powers that are not entirely obvious or controllable [1]. The *dawa* group contains an eclectic selection including medicines, battery acid, insecticides, and sorcery objects, as well as other substances and objects. The tendency for people in this study to group the medicines together could be helpful in designing health promotion interventions.

When it comes to their candidacy for sharing, the medicines were mixed with the other items. However, there was generally less agreement among participants concerning whether it was proper to share medicines compared to their opinions about sharing the other items. The uncertainty about sharing medicines combined with the inclination to think about medicines as a distinct category suggests that people might be open to interventions to change sharing behavior designed to apply broadly to the “medicine” category.

In general, medication adherence is sub-optimal with estimated non-adherence rates of 25% [34]. Attempts to improve adherence and the rational use of medicines have produced only modest results. Reviews of published studies and of 121 evaluable interventions from the World Health Organization’s database for developing and transitional countries indicate that improvements are often small and may not correlate with improved clinical outcomes [35], [36]. Discussions of the rational use of medicines generally do not even consider medicine sharing and its implications [6]. Strategies might most productively focus on consumers because those that depend heavily on professional education maybe less likely to produce large and durable changes [37].

The study findings suggest that specific groups of things in the first pile sort tended to have a similar candidacy for sharing as illustrated in Figure 3. The group of “personal items,” for example, generally had consistently lower shareability scores than the “medicines” group on that contour map. One could hypothesize that health promotion activity that successfully associates medicines with a group of things having a lower shareability score (such as establishing associations with the personal items) may decrease the medicines’ candidacy for sharing, as was seen with the inhaler.

Precedents for this type of created metaphoric association exist and are perhaps best studied in commercial advertising, for example the linking of drug-like pleasure sensations with food advertisements for children [38] and the well-studied effects of “Joe Camel” on smoking behavior [39]–[41]. Health promotion strategies that stress any similarities between medicines and personal items (for example, things kept in the bathroom or just for one person’s use) might offer an approach to decreasing medicine sharing.

It is noteworthy that the three HIV patients (who had received treatment literacy training to improve adherence) had all shared antiretroviral therapy when someone on the same medicine had a short-term need. Antiretroviral sharing occurred in the context of a temporary disruption in the national supply chain in which patients collaborated with health professionals in a re-distribution of available medicines and also occurred informally when individuals needed medicines for the short-term until they could get a refill.

While a conclusion drawn from Rouse’s work [42] is that “…doctors and patients were both unable to escape the logic within which non-compliance could lead to anything but poor health outcomes” [43], medicine sharing may not always be irrational or harm health. This sharing of antiretroviral therapy among people living with HIV/AIDS is an example of a rational sharing decision to support treatment compliance. In considering interventions related to medicine sharing, it may be well to recognize that the medication sharing behavior likely has deep cultural and relational roots, that some level of continuing medication sharing maybe inevitable, and that medication sharing could be resulting in some improved health outcomes.

This study had limitations. The pile sort results cannot be compared among individuals; the results represent a compendium of opinions – a “group cognition” – and can only be considered as a whole [29]. Accordingly, this study could not relate the personal characteristics of participants to the pile sort results. The study did not examine sharing behavior and factors such as younger age, being female, poverty, chronic pain, similarity of illness, and familial relationships, all of which other researchers have associated with sharing medicines [3]–[5], [7], [22]. The convenience sample had more women than men, possibly creating a bias toward the responses of women, the ones more likely to share medicines.

This study did not attempt to evaluate sharing as related to the social distance between the sharer and the receiver. Sharing behavior varies in different relationships, and social distance may be a primary factor in sharing, economic decisions, and drug commodification [2], [15], [18], [44], [45].

The study sample is relatively small. However, as the purpose was primarily descriptive, the loss of power is of less concern than in hypothesis testing studies where failure to reject a false null hypothesis could occur (a type II or β error). The convenience sample means that the study undoubtedly had unknown (and unknowable) biases. The biases limit the conclusions that can be drawn from the study, but they may not greatly influence the qualitative observations available from the data [46].

Most commercial medicines (except psychoactive pharmaceuticals and opiates) are available in the Dominican Republic without a prescription. This environment may be important when considering the results.

Medicines are universally popular and people share them. With appropriate interventions that acknowledge how people view and use medicines, a healthier standard of medicine sharing should be possible. This study has contributed to furthering the understanding of medicines from the perspective of those who obtain them, manage them, and sometimes pass them along.

## Supporting Information

File S1 **Supporting figures and tables.** Figure S1, Analogue scale used for pile sort constrained by candidacy for sharing. Figure S2, This association map shows the groupings of *materia medica* items in the free pile sort. Figure S3, Pile sort association map of items from the local *materia medica* with disease groups indicated. Table S1, Items in the pile sort of the *materia medica*, grouped according to health conditions they are understood to treat. Table S2, Individual item coordinates from free pile sort of 44 items. Table S3, Individual item coordinates from pile sort of 44 items constrained by shareability. Table S4, Individual item coordinates from free pile sort of items in *materia medica*.
(DOCX)
