# Supplementary material for: Sharing Medicine: The Candidacy of Medicines and Other Household Items for Sharing, Dominican Republic
Source: PLoS One. 2014 Jun 27;9(6):e101007. doi: 10.1371/journal.pone.0101007 (PMC4074169; doi:10.1371/journal.pone.0101007)
Supplement: File S1 — Supporting figures and tables. Figure S1, Analogue scale used for pile sort constrained by candidacy for sharing. Figure S2, This association map shows the groupings of materia medica items in the free pile sort. Figure S3, Pile sort association map of items from the local materia medica with disease groups indicated. Table S1, Items in the pile sort of the materia medica, grouped according to health conditions they are understood to treat. Table S2, Individual item coordinates from free pile sort of 44 items. Table S3, Individual item coordinates from pile sort of 44 items constrained by shareability. Table S4, Individual item coordinates from free pile sort of items in materia medica. (DOCX) [file pone.0101007.s001.docx]

**Supplementary Materials: File S1**

**Dohn, M. N. and Pilkington, H. Sharing medicine: the candidacy of medicines and other household items for sharing, Dominican Republic.**

**Figure S1.** Analogue scale used for pile sort constrained by candidacy for sharing.

| **0** | **1** | **2** | **3** |
| --- | --- | --- | --- |
| Assigned values for nonparametric analysis | | | |

Participants placed the cards from the pile sort on the scale according to their evaluation as to the candidacy of that item for sharing.

The values for each category on the scale were used to calculate the mean shareability score and its standard deviation for each item. The scores were used in the non-parametric analyses between the group of medicines and the other items.

Original size of the scale was approximately 13 by 5.5 inches.

| **Table S1.** Items in the pile sort of the *materia medica*, grouped according to health conditions they are understood to treat. | | | | | |
| --- | --- | --- | --- | --- | --- |
| **Headache** | **Hypertension** | **Intestinal Parasites** | **Anaemia** | **Tight**  **Breathing** | **Unclassified** |
| Hot hoe oranges  Hot purple agave  Crushed orange leaves  Acetaminophen  Diclofenac  Ibuprofen | Lemon balm  Garlic  Orange juice  Diuretic  Hypertension medicine  Matchwood tea  Peppers and pineapple juice | Lambsquarter  Purple basil  Green/water mint  Albendazole | Liver  Vitamin B complex injection  Black beans  Iton tablets  Cabbage  Beets | Generic liquid cold medicine  Coconut oil with herring  Shark oil  Oral inhaler  Pressured nebulizer treatment  Lily white onion | Small adhesive bandage  Vitamin capsules  Sal Andrews (patent medicine with antacids)  Hypodermic injection |

**Table S2.** Individual item coordinates from free pile sort of 44 items.

| **Item** | **X** | **Y** |  | **Item** | **X** | **Y** |
| --- | --- | --- | --- | --- | --- | --- |
| Cooking oil | -0.96 | 0.71 |  | Black beans | -0.88 | 0.59 |
| Acetaminophen | -0.50 | -0.87 |  | Water Mint | -0.96 | -0.11 |
| Garlic | -0.87 | 0.52 |  | Iron tablets | -0.55 | -1.07 |
| Purple basil | -1.03 | -0.03 |  | Ibuprofen | -0.41 | -1.03 |
| Albendazol | -0.50 | -0.81 |  | Soap | 0.58 | -0.27 |
| Pillow | 0.81 | -0.19 |  | Clothes washer | 0.36 | 0.66 |
| Cold medicine | -0.36 | -0.77 |  | Eye glasses | 1.34 | -0.18 |
| Rice | -0.92 | 0.59 |  | Blender | -0.04 | 0.92 |
| Ear phones | 1.01 | 0.86 |  | Hair comb | 0.92 | -0.20 |
| Sugar | -1.07 | 0.47 |  | Iron | 0.49 | 0.69 |
| Inhaler | -0.01 | -0.69 |  | Radio | 0.54 | 1.01 |
| Coffee maker | -0.23 | 0.79 |  | Beets | -0.93 | 0.43 |
| Purse | 0.69 | -0.07 |  | Cabbage | -1.08 | 0.66 |
| Music CD | 1.05 | 0.60 |  | Underwear | 0.89 | -0.38 |
| Mobile phone | 0.79 | 0.35 |  | Bed sheet | 0.56 | -0.14 |
| Tooth brush | 0.71 | -0.43 |  | Salt | -0.67 | 0.47 |
| Notebook | 1.46 | 0.20 |  | Antacid | -0.64 | -0.94 |
| Band aid | -0.11 | -0.70 |  | Towel | 0.92 | -0.33 |
| Diclofenac | -0.44 | -0.85 |  | Drinking glass | -0.25 | 0.70 |
| Diuretic | -0.39 | -1.05 |  | Candle | 0.48 | 0.26 |
| Spaghetti | -1.08 | 0.74 |  | Vitamins | -0.54 | -0.86 |
| Sun glasses | 1.09 | 0.01 |  | Shoes | 0.70 | -0.24 |

**Table S3.** Individual item coordinates from pile sort of 44 items constrained by shareability.

| **Item** | **X** | **Y** |  | **Item** | **X** | **Y** |
| --- | --- | --- | --- | --- | --- | --- |
| Cooking oil | 1.01 | 0.05 |  | Black beans | 1.14 | 0.24 |
| Acetaminophen | 0.30 | -0.89 |  | Water Mint | 1.19 | 0.16 |
| Garlic | 0.99 | 0.44 |  | Iron tablets | 0.11 | -0.96 |
| Purple basil | 1.04 | -0.04 |  | Ibuprofen | 0.31 | -0.91 |
| Albendazol | 0.15 | -0.98 |  | Soap | -1.07 | -0.31 |
| Pillow | -1.23 | 0.47 |  | Clothes washer | 0.19 | 0.94 |
| Cold medicine | 0.24 | -0.88 |  | Eye glasses | -1.10 | 0.06 |
| Rice | 0.94 | 0.06 |  | Blender | 0.29 | 0.77 |
| Ear phones | -0.39 | 0.69 |  | Hair comb | -1.16 | -0.12 |
| Sugar | 0.89 | 0.09 |  | Iron | 0.34 | 0.96 |
| Inhaler | -1.03 | -0.59 |  | Radio | 0.33 | 0.65 |
| Coffee maker | 0.27 | 1.00 |  | Beets | 1.03 | 0.21 |
| Purse | -0.65 | 0.40 |  | Cabbage | 1.05 | 0.25 |
| Music CD | 0.71 | 0.36 |  | Underwear | -1.27 | -0.23 |
| Mobile phone | -0.86 | 0.63 |  | Bed sheet | -1.06 | 0.39 |
| Tooth brush | -1.35 | -0.22 |  | Salt | 0.80 | 0.13 |
| Notebook | -0.06 | 0.13 |  | Antacid | 0.21 | -0.68 |
| Band aid | -0.01 | -0.62 |  | Towel | -1.34 | -0.05 |
| Diclofenac | 0.11 | -1.05 |  | Drinking glass | -0.47 | 0.15 |
| Diuretic | -0.15 | -1.03 |  | Candle | 0.32 | 0.46 |
| Spaghetti | 1.03 | 0.15 |  | Vitamins | 0.17 | -0.96 |
| Sun glasses | -0.98 | 0.16 |  | Shoes | -1.00 | 0.51 |

**Table S4.** Individual item coordinates from free pile sort of items in *materia medica*.

| **Item** | **X** | **Y** |  | **Item** | **X** | **Y** |
| --- | --- | --- | --- | --- | --- | --- |
| Acetaminophen | -0.71 | 0.48 |  | Liver | 1.22 | 0.34 |
| Garlic | 1.23 | 0.00 |  | Ibuprofen | -0.72 | 0.63 |
| Purple basil | 0.28 | -0.77 |  | Injection | -0.80 | 0.37 |
| Albendazol | -0.79 | 0.47 |  | Purple agave, warm | 0.07 | -0.72 |
| Cold medicine | -0.70 | 0.53 |  | Hoe oranges, warm | 0.09 | -0.88 |
| Lambsquarter | 0.10 | -0.68 |  | Crushed orange leaves | -0.07 | -0.67 |
| Inhaler | -0.97 | 0.27 |  | Orange juice | 0.94 | -0.90 |
| Lily white onion | 0.64 | -0.32 |  | Nebulizer | -0.94 | 0.33 |
| Coconut oil with herring | 0.60 | 0.04 |  | Pepper & pineapple juice | 0.98 | -1.12 |
| B complex vitamins | -0.53 | 0.52 |  | Hypertensive medicine | -0.63 | 0.58 |
| Band aid | -0.96 | 0.68 |  | Beets | 1.50 | -0.28 |
| Diclofenac | -0.73 | 0.56 |  | Cabbage | 1.63 | 0.22 |
| Diuretic | -0.70 | 0.69 |  | Antacid | -0.78 | 0.60 |
| Matchwood tea | 0.03 | -0.66 |  | Shark oil | -0.30 | 0.06 |
| Black beans | 1.69 | -0.04 |  | Lemon balm | 0.17 | -0.72 |
| Water Mint | 0.16 | -0.75 |  | Vitamins | -0.46 | 0.58 |
| Iron tablets | -0.52 | 0.53 |  |  |  |  |

**Figure S2.** This association map shows the groupings of *materia medica* items in the free pile sort (n = 30 sorts). Some items have been shifted to avoid overlapping text.

Diuretic

Band aid

Ibuprofen

Antacid Anti-hypertensive

Diclofenac Iron tablets Vitamins

Cold medicine B-complex vitamins

Acetaminophen

Albendazole Liver

Injection Cabbage

Nebulizer

Inhaler

Shark oil Coconut oil with herring

Garlic

Black beans

Beets

Lily white onion

Matchwood tea

Crushed orange leaves

Lambsquarter

Lemon balm

Purple agave

Water mint

Purple basil

Hoe oranges Orange juice

Peppers & pineapple juice

**Figure S3.** Pile sort association map of items from the local *materia medica* with disease groups indicated.

● Headache ▲ Intestinal parasites

■ Hypertension ♦ Anemia

∆ Tight breathing **+**  Unclassified
